# Predictors for in-hospital mortality from coronavirus disease 2019 (COVID-19) infection among adults aged 18–65 years

**DOI:** 10.1017/ice.2020.1390

**Published:** 2020-12-16

**Authors:** Ashish Bhargava, Mamta Sharma, Elisa Akagi, Susanna M. Szpunar, Louis Saravolatz

**Affiliations:** Ascension St John Hospital, Detroit, Michigan

As of October 13, 2020, at least 37,867,739 cases of coronavirus disease 2019 (COVID-19) had been identified worldwide, including ~7,804,699 cases in the United States.^1^ Early data from the United States showed that 20% of deaths occurred in patients aged 20–64 years and 80% occurred in patients aged ≥65 years.^2^ Understanding the risk factors associated with mortality among adults aged <65 years may identify vulnerable patients. The purpose of the study was to identify factors present at the time of hospital admission that predicted in-hospital mortality from COVID-19 among adults aged ≤65 years.

## Methods

We conducted a retrospective study at a 776-bed, tertiary-care center. The study was approved by the Ascension St John Hospital Institutional Review Board. We included adult patients with confirmed COVID-19 by positive real-time reverse-transcriptase-polymerase chain reaction (RT-PCR) assay of a nasopharyngeal swab for severe acute respiratory coronavirus virus 2 (SARS-CoV-2) from March 8 to June 14, 2020. Patient demographics, comorbid conditions, presenting symptoms, initial vital signs, admission laboratory and radiological findings, and outcome variables were extracted from the electronic medical records.

Statistical analyses were performed using SPSS version 27.0 software (IBM, Armonk, NY). Univariable analyses were conducted using the Student *t* test, the Mann-Whitney U test, and χ^2^ analysis. Variables that were significant or near-significant predictors of mortality (*P* < .09) were entered into a multivariable logistic regression model using a forward likelihood ratio algorithm. For comorbidities, the Charlson weighted index of comorbidity (CWIC) score was used instead of individual comorbid conditions.^3^ Results from the regression are reported as odds ratios with 95% confidence intervals. All reported *P* values are 2-sided.

## Results

In total, 265 hospitalized patients, ages 18–65 years, were included in this study. The mean age of the cohort was 50.4 years (SD, 10.7), 140 (52.8%) were male, and 226 (85.3%) were black/African-American. The mean body mass index of the cohort was 35.8 kg/m^2^ (SD, 9.3). The mean duration of symptoms prior to hospitalization was 5.8 days (SD, 3.8). Severe pneumonia was diagnosed in 49 patients (18.5%). Mechanical ventilation was required for 66 patients (24.9%). Overall, 214 patients (80.8%) improved clinically and survived to discharge.

The in-hospital case fatality rate (CFR) was 19.2% (51 of 265). Patients who died were significantly older (mean age, 53.4 years [SD, 9.4] vs 49.7 years [SD, 10.8]) than patients who survived (*P* = .03). Patients who died were significantly more likely to have hypertension, diabetes with chronic complications, hemiplegia, preexisting renal disease, liver disease, and a diagnosis of malignancy (Table 1). The prevalence of lymphocytopenia and thrombocytopenia were higher among patients who died than those who survived.

**Table 1.** Univariable Analysis of Predictors for in-Hospital Mortality Among Adults, Aged 18–65 Years From COVID-19 Infection at the Time of Hospital Admission

Characteristic	Survivors (*n*=214), No. (%)	Nonsurvivors (*n*=51), No. (%)	OR (95% CI)	*P* Value
**Sex, no. (%)**				
Male	113 (52.8)	27 (52.9)	1.0 (0.5–1.9)	.98
Female	101 (47.2)	24 (47.1)		
**Race, no. (%)**				
White	28 (13.1)	5 (9.8)		.80
Black	181 (84.6)	45 (88.2)		
Other	5 (2.3)	1 (2.0)		
BMI, mean ± SD	35.4 ± 8.8	37.7± 11.1		.12
**Admission source**				
Home	184 (86.01)	39 (76.5)	1.9 (0.9–4.0)	.10
Nursing facility	30 (14.0)	12 (28.6)		
**Insurance type**				
Commercial	108 (50.5)	11 (21.6)	3.7 (1.8–7.6)	<.0001
Public	106 (49.5)	40 (78.4)		
**Comorbidities, no. (%)**				
Myocardial infarction	7 (3.3)	3 (5.9)	1.8 (0.5–7.4)	.38
Congestive heart failure	20 (9.3)	3 (5.9)	0.6 (0.2–2.1)	.43
Peripheral vascular disease	10 (4.7)	3 (5.9)	1.3 (0.3–4.8)	.72
Cerebrovascular disease	21 (9.9)	2 (3.9)	0.4 (0.1–1.6)	.18
Dementia	11 (5.1)	4 (7.8)	1.6 (0.5–5.2)	.45
Chronic pulmonary disease	41 (19.2)	14 (27.5)	1.6 (0.8–3.2)	.19
Connective tissue disease	2 (0.9)	1 (2.0)	2.1 (0.2–23.8)	.53
Peptic ulcer disease	7 (3.3)	1 (2.0)	0.6 (0.07–4.9)	.62
Diabetes without chronic complications	53 (24.8)	11 (17.2)	0.8 (0.4–1.7)	.63
Diabetes with chronic complications	15 (7.0)	13 (25.5)	4.5 (2.0–10.3)	<.0001
Hemiplegia	7 (3.3)	9 (17.6)	6.3 (2.2–18.0)	<.0001
Pre-existing renal disease	28 (13.1)	13 (25.5)	2.3 (1.1–4.8)	.03
Any malignancy	2 (0.9)	3 (5.9)	6.6 (1.1–40.7)	.02
Metastatic solid tumor	0 (0.0)	1 (2.0)	…	…
Mild liver disease	1 (0.5)	2 (3.9)	8.7 (0.8–105.3)	.04
Moderate-severe liver disease	1 (0.5)	2 (3.9)	8.7 (0.8–97.8)	.04
AIDS	3 (1.4)	0 (0.0)	…	…
Median CWIC (25^th^–75^th^)	0.0 (0,1)	1.0 (0–3.0)		<.0001
Hypertension	129 (60.3)	42 (82.4)	3.1 (1.4–6.6)	.003
Current tobacco smoker	17 (7.9)	5 (10.0)	1.3 (0.5–3.7)	.67
Obesity	149 (71.0)	39 (76.5)	1.3 (0.7–2.7)	.43
History of sick contact	70 (33.8)	1615 (32.0)	0.9 (0.5–1.8)	.81
**Symptoms, no. (%)**				
Fever	147 (69.0)	33 (66.0)	0.9 (0.5–1.7)	.68
Shortness of breath	156 (73.5)	41 (82.0)	1.7 (0.8–3.6)	.20
Cough	160 (75.5)	34 (69.4)	0.7 (0.4–1.5)	.38
Loss of taste	35 (16.7)	5 (10.4)	0.6 (0.2–1.6)	.28
**Vitals on admission**				
Systolic BP, mean ± SD	135.6 ± 23.7	136.0 ± 29.3		.92
Diastolic BP, mean ± SD	76.7 ± 15.9	76.2 ± 1729		.85
Heart rate, mean ± SD	104.1 ± 21.2	112.7 ± 21.0		.02
Respiratory rate, mean ± SD	22.8 ± 7.0	27.1 ± 8.4		<.0001
Fever, no. (%)	88 (41.1)	21 (41.2)	1.0 (0.5–1.9)	.99
Oxygen saturation, mean ± SD	0.94 ± 0.06	0.91 ± 0.09		.001
**qSOFA score**s				<.0001
0	93 (43.5)	10 (19.6)		
1	105 (49.1)	28 (54.9)		
2	14 (6.5)	10 (19.6)		
3	2 (0.9)	3 (5.9)		
Abnormal chest x-ray on admission, no. (%)	152 (71.0)	42 (82.4)	1.9 (0.9–4.1)	.10
**Laboratory findings on admission, no. (%)**				
Leukopenia	22 (10.3)	6 (11.8)	1.2 (0.5–3.0)	.76
Lymphocytopenia (<1.0×10^9^/L)	84 (39.3)	28 (54.9)	1.9 (1.0–3.5)	.04
Thrombocytopenia (<150×10^9^/L)	29 (13.6)	19 (37.3)	3.8 (1.9–7.5)	<.0001
Elevated AST (>40 U/L)	110 (53.9)	33 (71.7)	2.2 (1.1–4.4)	.03
Elevated ALT (>40 U/L)	81 (38.8)	15 (30.6)	0.7 (0.4–1.4)	.29
Low serum protein (<6.2 gm/dL)	6 (2.9)	5 (10.2)	3.8 (1.1–13.1)	.02
Low serum albumin (<3.5 gm/dL)	60 (28.8)	26 (53.1)	2.8 (1.5–5.3)	.001
Elevated creatinine (from baseline)	55 (26.6)	27 (57.4)	3.7 (1.9–7.2)	<.0001
**Positive blood culture on admission**	5 (5.2)	3 (12.0)	2.5 (0.6–11.2)	.22
**Clinical diagnosis, no. (%)**				
Uncomplicated illness	103 (48.1)	17 (33.3)		.008
Mild pneumonia	79 (36.9)	17 (33.3)		
Severe pneumonia	32 (15.0)	17 (33.3)		

Note. OR, odds ratio; CI, confidence interval; BMI, body mass index; SD, standard deviation; AIDS, acquired immunodeficiency syndrome; CWIC, Charlson weighted index of comorbidity; BP, blood pressure; AST, aspartate aminotransferase; ALT, alanine aminotransferase; CRP, C-reactive protein; qSOFA, quick sepsis-related organ-failure assessment.

The Quick Sequential Organ Failure Assessment (qSOFA) score (0–3) is composed of 3 clinical parameters with 1 point allotted to each of these parameters: systolic blood pressure ≤100 mm Hg, respiratory rate ≥22 breaths per minute, and altered mental status. For patients with qSOFA scores of 0, 1, 2, and 3 at the time of hospital admission, the mortality rates were 9.7%, 21.1%, 41.7%, and 60%, respectively (*P* < .0001).

The final multivariable logistic regression model included 4 variables that predicted increased odds of death in patients with COVID-19 infection: Charlson score, presence of hypertension, qSOFA, and thrombocytopenia at the time of hospital admission (Table 2).

**Table 2.** Multivariable Analysis of Predictors for In-Hospital Mortality Among Adults, Aged 18–65 Years From COVID-19 Infection at the Time of Hospital Admission

Variables	OR (95% CI)	*P* Value
CWIC score	1.3 (1.1–1.6)	<.0001
Thrombocytopenia	3.5 (1.5–8.0)	.003
Hypertension	2.8 (1.1–6.8)	.03
qSOFA score of 1^a^	3.1 (1.3–7.8)	.02
qSOFA score of 2^a^	6.2 (1.8–20.9)	.003
qSOFA score of 3^a^	15.2 (1.7–135.2)	.02

Note. OR, odds ratio; CI, confidence interval; CWIC, Charlson weighted index of comorbidity; qSOFA, quick sepsis-related organ-failure assessment. qSOFA 1 has a score of 1, qSOFA 2 has a score of 2, and qSOFA 3 has a score of 3.

a Compared to a qSOFA score of zero.

## Discussion

In our study, the CFR among patients aged ≤65 years was 19.2%, which is similar to previous US studies, in which mortality ranged from 8.3 % to 22.7%.^4^ The qSOFA score was introduced by the Sepsis-3 task force as a tool to assist in the early identification of patients at risk of sepsis. Patients with a qSOFA score of 2 or higher had a mortality rate of 24% compared to 3% among patients with a qSOFA score of <2.^5^ In our study, a qSOFA score of 2 was associated with a mortality rate of 41.7%.

Thrombocytopenia in critically ill patients usually suggests serious organ malfunction or physiologic decompensation, often evolving toward disseminated intravascular coagulation. In a meta-analysis, a lower platelet count was encountered in nonsurviving COVID-19 patients.^6^ In our study, 37.3% of nonsurvivors had thrombocytopenia on presentation compared to 13.6% of survivors.

Similar to clinical studies, we found that a higher Charlson score was an independent predictor of mortality.^7-9^ In terms of individual comorbidities, hypertension, diabetes with chronic complications, hemiplegia, renal disease, malignancy, and liver disease, were associated with mortality. Previous studies from China have reported mortality in patients with comorbidities of 10.5% for cardiovascular disease, 7.3% for diabetes, 6.3% for chronic respiratory disease, 6% for hypertension, and 5.6% for cancer.^7^ Previously, we reported that acute or pre-existing renal disease was an independent predictor for severe COVID-19 infection.^9^ A study of adults aged 18–34 years identified significantly higher mortality among patients with hypertension.^10^

This study has several limitations. It was limited by the sample size. This study was a single institution study of admitted patients which makes generalization difficult. Because of the retrospective design, certain laboratory results were sometimes unavailable on admission, including lactate dehydrogenase, D-dimer, and serum ferritin. Patients with chronic lung disease and conditions associated with immunosuppression were only a small percentage among hospitalized patients. Therefore, the role of some of these variables in predicting mortality from COVID-19 could have been underestimated.

In conclusion, calculation of the qSOFA score bedside at the time of admission can predict mortality among COVID-19 patients aged ≤65 years. These findings can be applied globally, including resource-limited countries. Subsequent research involving multiple study sites and with a larger database can further validate the findings of our study.
